# The Type IVa Pilus Machinery Is Recruited to Sites of Future Cell Division

**DOI:** 10.1128/mBio.02103-16

**Published:** 2017-01-31

**Authors:** Tyson Carter, Ryan N. C. Buensuceso, Stephanie Tammam, Ryan P. Lamers, Hanjeong Harvey, P. Lynne Howell, Lori L. Burrows

**Affiliations:** a Department of Biochemistry and Biomedical Sciences and Michael G. DeGroote Institute for Infectious Disease Research, McMaster University, Hamilton, Ontario, Canada; b Program in Molecular Structure & Function, The Hospital for Sick Children, Toronto, Ontario, Canada; c Department of Biochemistry, University of Toronto, Toronto, Ontario, Canada; Max Planck Institute for Terrestrial Microbiology

## Abstract

Type IVa pili (T4aP) are ubiquitous microbial appendages used for adherence, twitching motility, DNA uptake, and electron transfer. Many of these functions depend on dynamic assembly and disassembly of the pilus by a megadalton-sized, cell envelope-spanning protein complex located at the poles of rod-shaped bacteria. How the T4aP assembly complex becomes integrated into the cell envelope in the absence of dedicated peptidoglycan (PG) hydrolases is unknown. After ruling out the potential involvement of housekeeping PG hydrolases in the installation of the T4aP machinery in *Pseudomonas aeruginosa*, we discovered that key components of inner (PilMNOP) and outer (PilQ) membrane subcomplexes are recruited to future sites of cell division. Midcell recruitment of a fluorescently tagged alignment subcomplex component, mCherry-PilO, depended on PilQ secretin monomers—specifically, their N-terminal PG-binding AMIN domains. PilP, which connects PilO to PilQ, was required for recruitment, while PilM, which is structurally similar to divisome component FtsA, was not. Recruitment preceded secretin oligomerization in the outer membrane, as loss of the PilQ pilotin PilF had no effect on localization. These results were confirmed in cells chemically blocked for cell division prior to outer membrane invagination. The hub protein FimV and a component of the polar organelle coordinator complex—PocA—were independently required for midcell recruitment of PilO and PilQ. Together, these data suggest an integrated, energy-efficient strategy for the targeting and preinstallation—rather than retrofitting—of the T4aP system into nascent poles, without the need for dedicated PG-remodeling enzymes.

## IMPORTANCE

The peptidoglycan (PG) layer of bacterial cell envelopes has limited porosity, representing a physical barrier to the insertion of large protein complexes involved in secretion and motility. Many systems include dedicated PG hydrolase components that create space for their insertion, but the ubiquitous type IVa pilus (T4aP) system lacks such an enzyme. Instead, we found that components of the T4aP system are recruited to future sites of cell division, where they could be incorporated into the cell envelope during the formation of new poles, eliminating the need for PG hydrolases. Targeting depends on the presence of septal PG-binding motifs in specific components, as removal of those motifs causes delocalization. This preinstallation strategy for the T4aP assembly system would ensure that both daughter cells are poised to extrude pili from new poles as soon as they separate from one another.

## INTRODUCTION

The cell envelopes of most Gram-negative bacteria are composed of an inner membrane (IM), a peptidoglycan (PG) layer found in the periplasm, and an outer membrane (OM). A variety of large protein complexes, including motility machines, secretion systems, and efflux pumps, span all of these layers (1), but in many cases, the mechanisms used to integrate these systems into the cell envelope remain uncharacterized. Often they are integrated at specific locations in the cell, such as the poles, which is important for their function.

The machinery that assembles and disassembles type IVa pili (T4aP) is among the most common cell envelope-spanning complexes found in Gram-negative bacteria. T4aP are long, thin protein fibers used for adherence, biofilm formation, and a flagellum-independent form of motility known as twitching, defined as repeated cycles of pilus extension, adherence to a surface, and retraction of the pilus fiber (2, 3). *Pseudomonas aeruginosa* T4aP are important for surface mechanosensing and consequent upregulation of virulence factor expression (4) and for the delivery of toxins by other secretion systems (5, 6). In the absence of T4aP, *P. aeruginosa* has reduced pathogenicity (5).

The T4aP machinery is composed of four subcomplexes that together form a dynamic cylindrical nanomachine (3, 7, 8). The IM motor and alignment subcomplexes form the platform for assembly and disassembly of the pilus and interact directly with the secretin that allows for pilus extrusion through the OM. The OM lipoprotein PilF promotes formation of the secretin, a highly stable oligomer of 14 PilQ subunits that form a gated channel in the OM (9, 10). The N terminus of each PilQ monomer contains two tandem amidase N-terminal (AMIN) domains involved in PG binding (11–13). The alignment subcomplex composed of PilMNOP connects the motor subcomplex with the secretin and participates in pilus extension and retraction (14–17). PilM is a cytoplasmic, actin-like protein that clamps tightly onto the short amino terminus of the IM protein PilN, which in turn forms heterodimers with the IM protein PilO (14, 18, 19). The IM lipoprotein PilP interacts with PilNO heterodimers via its long unstructured N terminus and with the periplasmic N0 domain of PilQ via its C-terminal homology region (HR) domain, completing the transenvelope complex (17, 20).

T4aP are usually located at the poles of rod-shaped cells, which promotes adherence by minimizing the surface area and thus electrostatic repulsion (3). How the multiprotein T4aP system is initially targeted to and integrated into the poles of *P. aeruginosa* cells is not well understood. Type III secretion systems (T3SSs) and T4SSs have dedicated PG-remodeling enzymes that allow for retrofitting of the complexes into the cell envelope (1). There are no such enzymes associated with the T4aP system, suggesting that it uses an alternate pathway for cell envelope integration. Here we used fluorescent fusions to an informative alignment subcomplex component, PilO, and to the secretin monomer PilQ to track and quantify their localization in the *P. aeruginosa* wild-type (WT) and mutant backgrounds. The results show that PilO and associated components are recruited to future sites of cell division by the concerted action of at least three pathways. We propose that the T4aP machinery is preinstalled during the formation of nascent cell poles, a strategy that could eliminate the need for dedicated cell wall-processing enzymes.

## RESULTS

### mCherry-PilO localizes to cell poles and future cell division sites.

**FIG 1:**
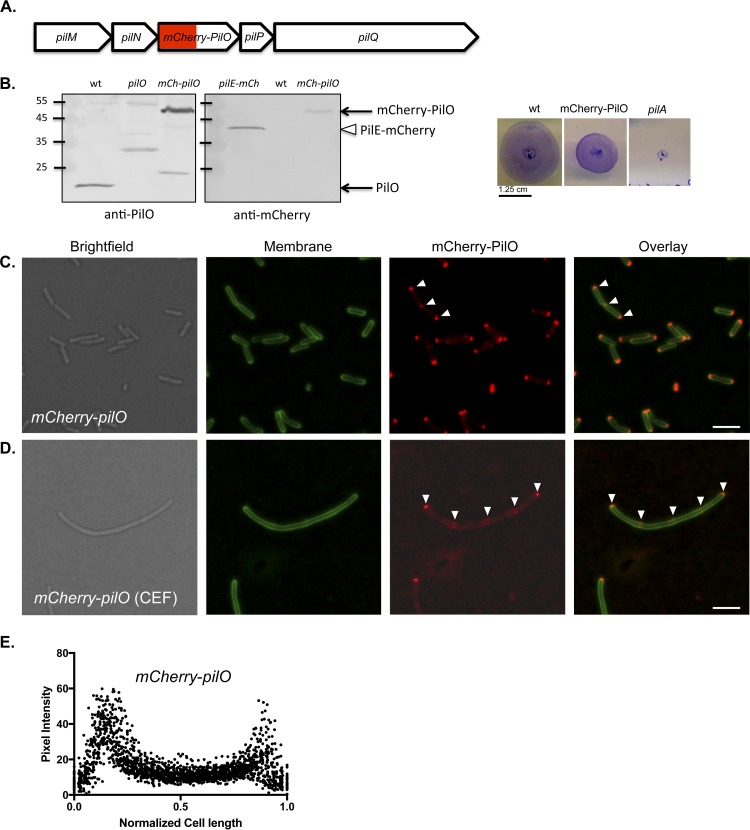
mCherry-PilO localizes to cell poles and future sites of cell division in *P. aeruginosa*. (A) Map of the T4aP alignment subcomplex operon showing mCherry-*pilO* integration. (B) The mCherry-PilO fusion was stable (~48-kDa product recognized by both anti-PilO and anti-mCherry antibodies; a plasmid-encoded PilE-mCherry fusion [open triangle] was used as a positive control for the latter [61]) and functional for twitching motility. The values to the left indicate mass in kilodaltons. (C) mCherry-PilO localized to the poles of WT cells and to the septum in late-stage dividing cells. (D) When cells were filamented with 40 μg/ml cefsulodin (CEF), mCherry-PilO localized to the poles and to regularly spaced foci (arrowheads). Scale bar, 3 μm. (E) mCherry pixel intensity in 50 untreated cells after normalization of length to 1 as described in Materials and Methods.

When selecting components of the T4aP machinery to track with fluorescent fusions, we considered the known interactions and stoichiometry of these proteins and how a fusion might affect function. PilM is cytoplasmic but has multiple interaction partners (14, 19, 20), including PilN, whose cytoplasmic N terminus binds to a groove on PilM; thus, we excluded both as candidates. PilN interacts along most of its remaining length with PilO (15, 21), but PilO’s short cytoplasmic N terminus is poorly conserved among T4aP-expressing species (16), supporting the observation that it appears to have no cytoplasmic interaction partners. Therefore, PilO was selected as a fusion candidate for localization of the alignment subcomplex. To maintain the physiological expression levels and stoichiometry that are important for function (14), a *pilO* construct encoding an in-frame fusion of mCherry to the N terminus of PilO was used to replace the WT version at the native *pilO* locus (Fig. 1A). The stability and functionality of the fusion protein were verified by Western blotting with anti-PilO and anti-mCherry antisera and assessment of twitching motility, respectively (Fig. 1B).

mCherry-PilO localized to both poles, as well as to the midpoint, of cells undergoing division (Fig. 1C). To more clearly visualize the predivisional localization pattern of mCherry-PilO, cells were treated with subinhibitory levels of cefsulodin, which inhibits PBP3 (FtsI), a late-stage PG transpeptidase, leading to division arrest and filamentation (22). In filamented cells, mCherry-PilO localized to the poles and to regularly spaced foci, suggestive of recruitment to future sites of cell division (Fig. 1D). Quantification of mCherry-PilO localization in WT cells (Fig. 1E) confirmed its predominantly bipolar localization.

**FIG 2:**
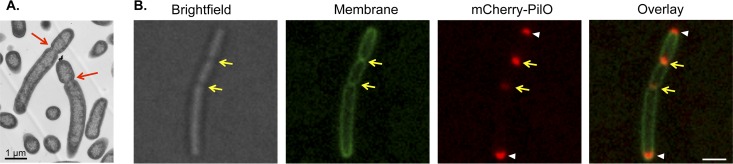
mCherry-PilO localizes to aberrantly localized septa in a *minC* mutant. (A) Transmission electron micrograph of a *minC* mutant of *P. aeruginosa* strain PAK. In the absence of *minC*, cells had aberrantly located division sites (red arrows). Scale bar, 1 μm. (B) mCherry-PilO polar foci are indicated by white arrowheads, while foci localized to aberrantly placed septa are indicated by yellow arrows.

To provide further evidence that mCherry-PilO was recruited to sites of cell division, the *minC* gene, which encodes a key component of the oscillating Min system required for inhibition of FtsZ polymerization at nonmidcell locations (23, 24), was deleted from the mCherry-PilO strain. Cells lacking MinC exhibit aberrantly localized septa, producing minicells because of unequal cell division (Fig. 2A). As predicted, the mCherry-PilO fusion continued to localize to polar and septal sites despite their aberrant placement (Fig. 2B). Together, these data suggest that when expressed under physiological conditions, PilO—and, by inference, its interaction partners PilMN and PilP (20)—is recruited to future sites of cell division.

### PilQ monomers are required for T4aP alignment subcomplex localization.

**FIG 3:**
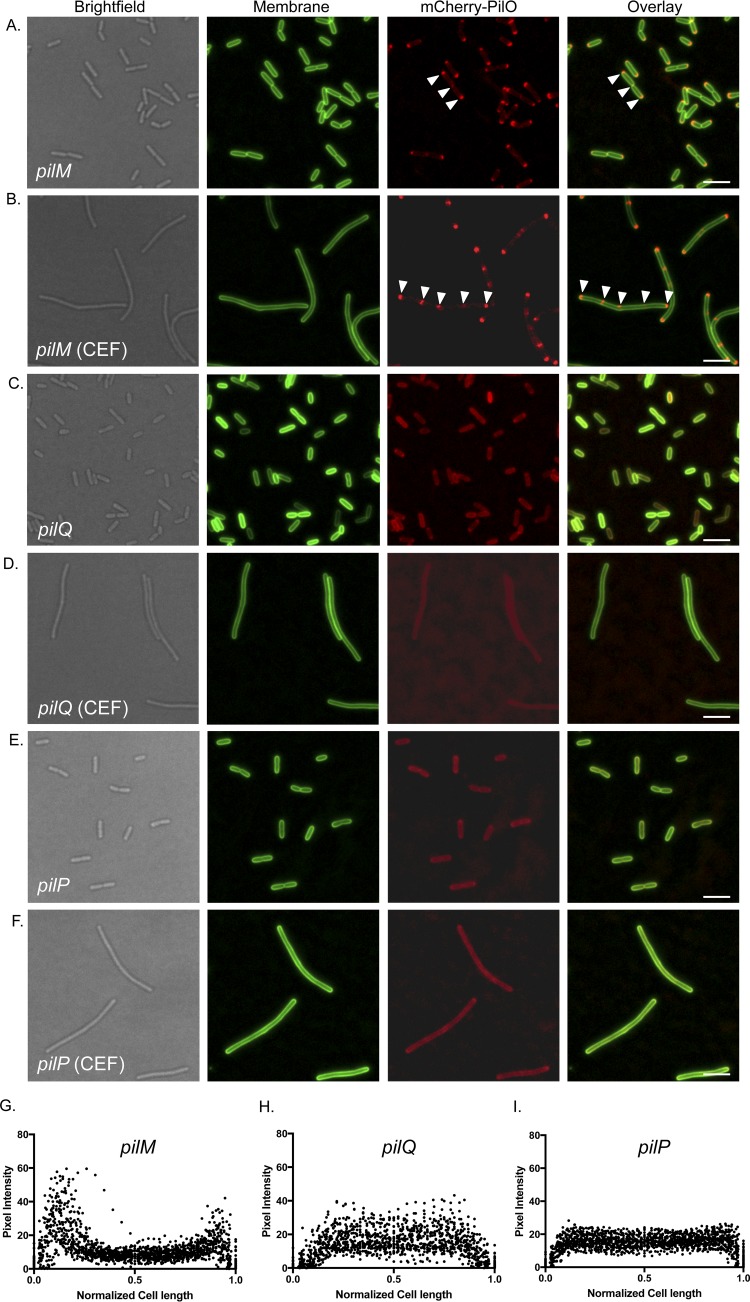
Polar localization of T4aP alignment subcomplex requires PilQ. In the absence of PilM, mCherry-PilO was localized to poles and septa in untreated cells (A) and poles and regularly spaced foci in cells treated with 40 μg/ml cefsulodin (CEF) (B). In the absence of PilQ, mCherry-PilO was delocalized in both untreated (C) and CEF-filamented (D) cells. Similarly, in the absence of PilP, mCherry-PilO was delocalized in untreated (E) and CEF-treated (F) cells. The last three panels show the quantification of mCherry pixel intensity in the absence of *pilM* (G), *pilQ* (H), or *pilP*, (I) in 50 untreated cells after normalization of length to 1 as described in Materials and Methods. Scale bars, 3 μm.

To define the mechanism of recruitment of the T4aP alignment subcomplex to sites of future cell division, we first examined the effects of deleting specific subcomplex components. The cytoplasmic member of the alignment subcomplex, PilM, has pronounced structural similarity (Cα root mean square deviation of 1.9 Å over 257 residues) to the early divisome protein, FtsA, a peripheral IM protein whose interaction with FtsZ tethers the latter to the membrane (18, 19). On the basis of its structural mimicry of FtsA and its peripheral membrane localization via the binding to PilN’s amino terminus, we previously hypothesized (25) that PilM—and thus the alignment subcomplex—might be recruited to a midcell position through interactions with the FtsZ ring. However, mCherry-PilO exhibited polar and midcell localization in a *pilM* mutant (Fig. 3A and B), ruling out PilM as a driver of recruitment.

The PilMNOP alignment subcomplex connects to the secretin via interaction of the C-terminal HR domain of PilP with the periplasmic N0 domain of PilQ (20). Preceding the N0 domain in *P. aeruginosa* PilQ are two tandem AMIN domains, predicted to bind septal PG (11–13). To test their PG-binding ability, we cloned, expressed, and purified three fragments of PilQ’s N-terminal region (the AMIN domains alone; the N0-N1 domains alone; and all four domains together) and performed pulldown assays with purified *P. aeruginosa* PG (see Text S1 and Fig. S1 in the supplemental material). In the absence of PG, all fragments remained in the soluble fraction; however, when insoluble PG was present, only those fragments containing the AMIN domains were also found in the insoluble fraction, indicating that they bind PG.

10.1128/mBio.02103-16.1 TEXT S1  Experimental details of the PG pulldown of PilQ fragments (see Fig. S1) and details of the quantification of *pocA* mutant and complemented *pocA* mutant cell lengths (see Fig. S4). Download TEXT S1, DOCX file, 0.1 MB. Copyright © 2017 Carter et al. 2017 Carter et al. This content is distributed under the terms of the Creative Commons Attribution 4.0 International license.

10.1128/mBio.02103-16.2 FIG S1  The AMIN domains of PilQ bind PG. Periplasmic fragments of PilQ corresponding to AMIN plus N0 and N1 (PilQ_24-445_), AMIN only (PilQ_24-280_), or N0-N1 only (PilQ_281-445_) were purified and incubated with purified *P. aeruginosa* PG as described in Text S1. Only those fragments containing the AMIN domains were recovered in the PG-bound fraction. Download FIG S1, PDF file, 0.1 MB. Copyright © 2017 Carter et al. 2017 Carter et al. This content is distributed under the terms of the Creative Commons Attribution 4.0 International license.

Since *pilMNOPQ* are expressed from a single operon, we hypothesized that recruitment of PilMNOP to sites of cell division might occur through a series of PilMNOP-PilQ-septal PG interactions. We first confirmed that PilQ-mCherry was localized to the poles and to regularly spaced foci in filamented cells, a pattern similar to that of mCherry-PilO (see Fig. S2). When we deleted *pilQ*, mCherry-PilO became delocalized (Fig. 3C and D). To confirm that loss of mCherry-PilO localization in the *pilQ* mutant strain was indirectly due to loss of PilP-PilQ interactions, *pilP* was deleted from the mCherry-PilO strain. In the *pilP* mutant, mCherry-PilO was delocalized (Fig. 3F), supporting the hypothesis that recruitment of PilQ to a midcell position is the primary event and the remaining alignment subcomplex components are recruited via their interaction with PilP and its interaction with PilQ.

10.1128/mBio.02103-16.3 FIG S2  PilQ-mCherry is delocalized in the *fimV* and *pocA* backgrounds but not in the *pilF* background. PilQ-mCherry was expressed in *trans* from the leaky promoter of the pBADGr vector without arabinose induction. Cells were treated with cefsulodin to inhibit division, making it easier to see changes in PilQ localization. (A) The PilQ-mCherry fusion localized to the poles and regularly spaced foci in a *pilQ* mutant. (B) In the absence of the pilotin PilF, PilQ-mCherry monomers in the IM localized to the poles and regularly spaced foci. (C) PilQ-mCherry was delocalized in a *fimV* mutant. (D) In a strain expressing FimV lacking its LysM domain, PilQ-mCherry was partly delocalized, as polar foci were still present. (E) In the absence of PocA, PilQ-mCherry delocalization is similar to that observed in the *fimV* background. Cell membranes were stained with FM1-43FX dye (in 10 μg/ml dye solution). Cells were stab inoculated into 1% LB agar in chambered glass coverslips, incubated at 37°C, and imaged with a 63× oil immersion objective on an EVOS FL digital imaging system. Images were processed with Fiji. Scale bars, 3 μm. Download FIG S2, PDF file, 0.9 MB. Copyright © 2017 Carter et al. 2017 Carter et al. This content is distributed under the terms of the Creative Commons Attribution 4.0 International license.

**FIG 4:**
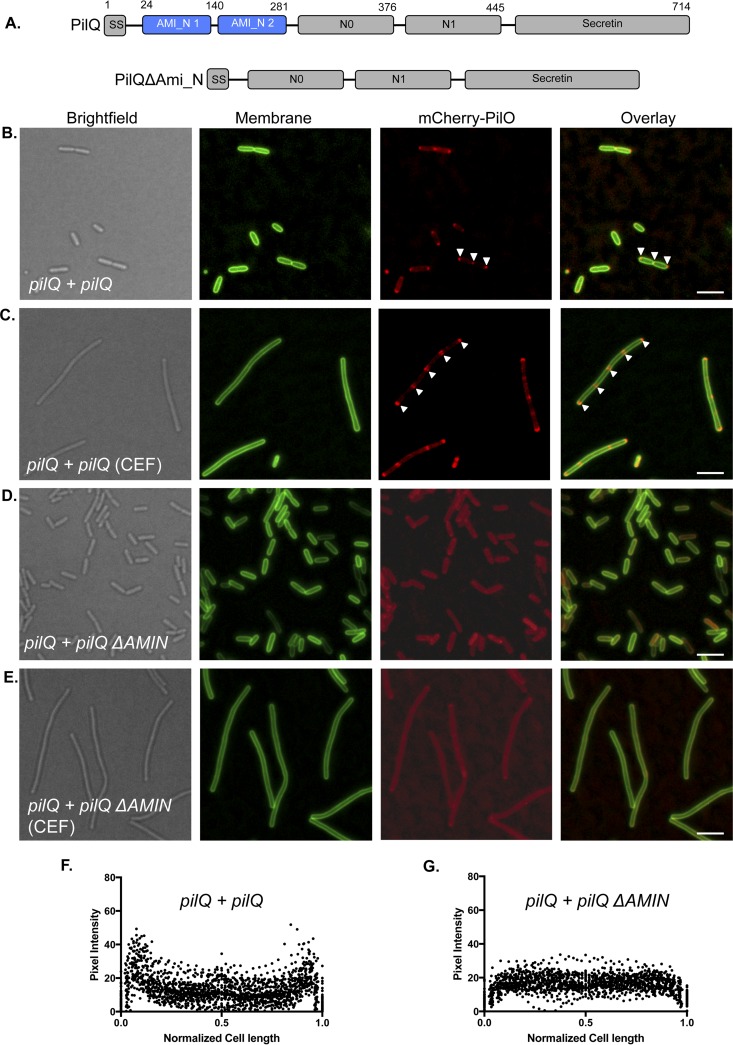
The AMIN domains of PilQ are required for polar localization of mCherry-PilO. (A) Domain map of PilQ showing its PG-binding AMIN domains in blue. Complementation of a *pilQ* mutant expressing mCherry-PilO with full-length *pilQ* (*pilQ + pilQ*) reestablished its polar and septal localization (white arrowheads) in untreated cells (B) and localization to the poles and regularly spaced foci in cells treated with 40 μg/ml cefsulodin (CEF) (C). When the same mutant was complemented with a truncated version of *pilQ* that encodes only N0-N1 and the secretin domains (*pilQ* + *pilQ* Δ*AMIN*), mCherry-PilO was delocalized in both untreated (D) and CEF-treated (E) cells. The last two panels show the quantification of mCherry pixel intensity in the presence of PilQ (F) or PilQ lacking its AMIN domains (G) in 50 untreated cells after the normalization of length to 1 as described in Materials and Methods. Scale bars, 3 μm.

To test whether its AMIN domains were responsible for the localization of PilQ and its interaction partners to midcell and polar positions, we complemented a *pilQ* mutant expressing mCherry-PilO with full-length *pilQ* or a construct expressing only the N0-N1 and secretin domains, previously shown to form stable but nonfunctional secretin oligomers (Fig. 4A) (9). Full-length PilQ restored the WT pattern of mCherry-PilO localization (Fig. 4B and C), but AMIN-deficient PilQ did not (Fig. 4D and E). These data show that the localization of T4aP assembly components is dependent upon PilQ’s AMIN domains, which interact with PG (see Fig. S1).

### Localization of mCherry-PilO precedes PilQ oligomerization.

**FIG 5:**
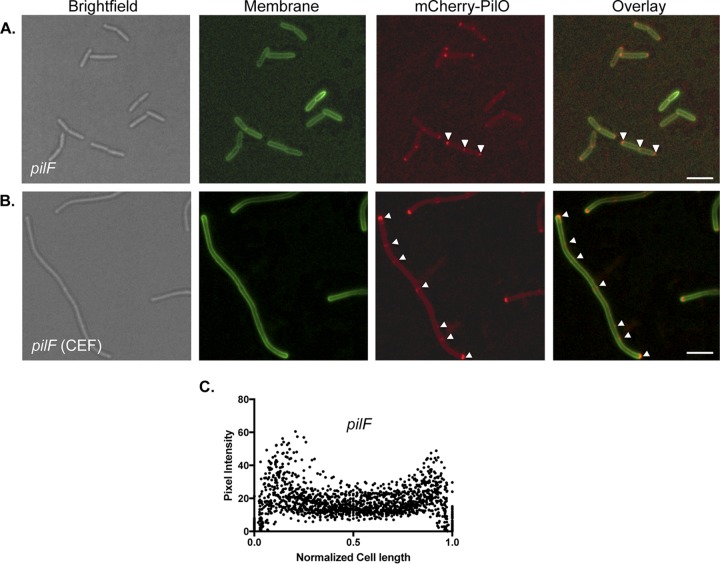
mCherry-PilO is polarly localized in the absence of *pilF*. (A) mCherry-PilO localized to the poles and septa of late-stage dividing cells, marked by white arrowheads. (B) When cells were filamented with cefsulodin, mCherry-PilO localized to the poles and to foci (arrows). Scale bars, 3 μm. Panel C shows the quantification of mCherry pixel intensity in the *pilF* background in 50 untreated cells after the normalization of length to 1 as described in Materials and Methods.

The *Myxococcus xanthus* T4aP system was proposed to assemble in an “outside-in” manner, with PilMNOP alignment subcomplexes docking onto the PilQ secretin after its assembly in the OM (26). Those data are not consistent with our observation that mCherry-PilO localized to regularly spaced foci in chemically filamented *P. aeruginosa* cells (Fig. 1), where the OM is not yet invaginated due to arrest of cell division. To test whether secretin oligomerization is a necessary prerequisite for recruitment, we examined the localization of PilQ-mCherry in mutants lacking the OM lipoprotein PilF. In the absence of PilF, PilQ remains in a monomeric state in the IM (9), but its localization pattern was unchanged compared to that in the WT (see Fig. S2A and B). Similarly, the localization of mCherry-PilO in the *pilF* background resembled that in the WT (Fig. 5). These data confirm that the localization of T4aP components to a midcell position—while PilQ-dependent—precedes secretin oligomerization.

### Additional factors modulate T4aP assembly machinery localization.

**FIG 6:**
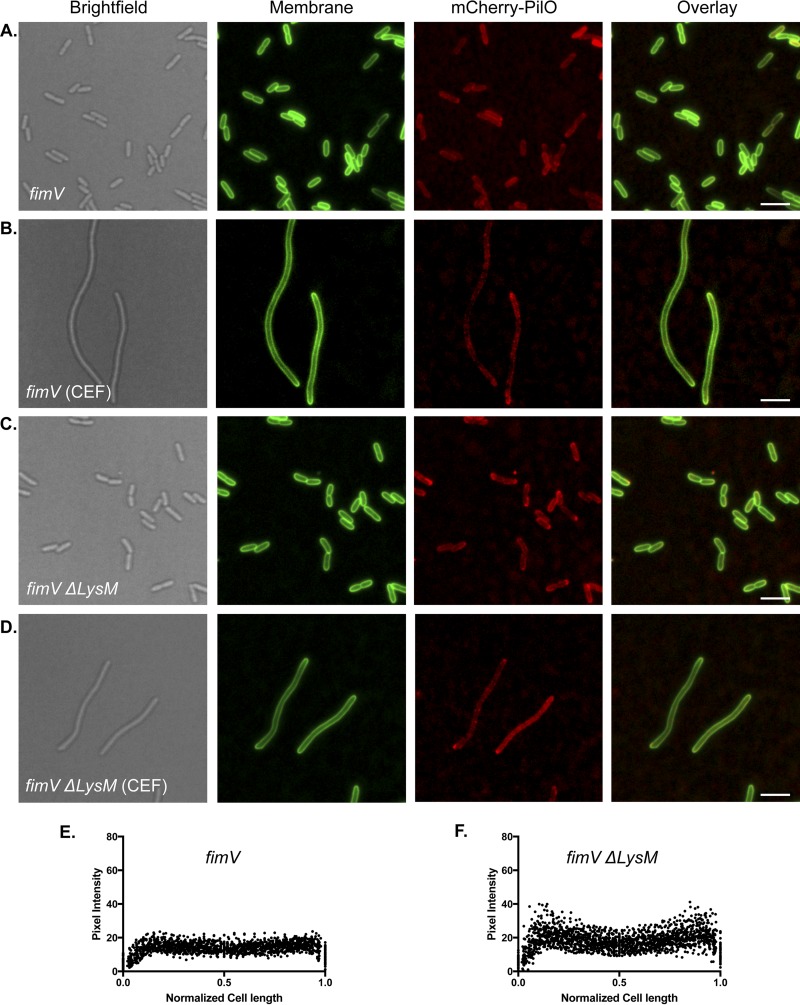
FimV is required for polar localization of mCherry-PilO. In the absence of FimV, mCherry-PilO was delocalized in untreated cells (A) and cells treated with cefsulodin (B). In cells expressing a version of FimV with an in-frame deletion of its PG-binding domain (LysM), mCherry-PilO was delocalized in untreated (C) and antibiotic-treated (D) cells. The last two panels show the quantification of mCherry pixel intensity in the absence of FimV (F) or in the strain expressing FimVΔ*LysM* (G) in 50 untreated cells after the normalization of length to 1 as described in Materials and Methods. Scale bars, 3 μm.

FimV is a T4aP-associated protein that is required for twitching motility and has a highly conserved PG-binding LysM motif in its periplasmic N-terminal domain (27, 28). We showed previously (28) that PilQ multimer formation is reduced in a *fimV* mutant, which made FimV of interest for this study. In the absence of FimV, mCherry-PilO was delocalized (Fig. 6A and B), as was PilQ-mCherry (see Fig. S2C). We previously generated a strain expressing a variant of FimV with an in-frame deletion of its LysM motif (28). mCherry-PilO (Fig. 6C and D) was delocalized in the *fimV* Δ*lysM* mutant strain, as was most of PilQ-mCherry (see Fig. S2D). These data suggest that interactions of both PilQ and FimV with PG are important steps in the localization process and that polar localization of PilQ depends in part on FimV.

During a mutant screen for factors required for correct localization of *P. aeruginosa*’s single polar flagellum, Cowles and colleagues (29) identified a putative protein complex that they named PocAB-TonB3 for “polar organelle coordinator.” Loss of TonB3 had previously been shown to affect twitching motility (30), but the mechanism was unknown. All three components were required for the expression and/or polar localization of T4aP. *pocA* mutants made a few nonpolar pili, while *pocB* and *tonB3* mutants failed to produce pili. The mechanism by which this system controls polar localization of motility organelles remains unclear, since the Poc proteins themselves are not localized to the poles (29).

**FIG 7:**
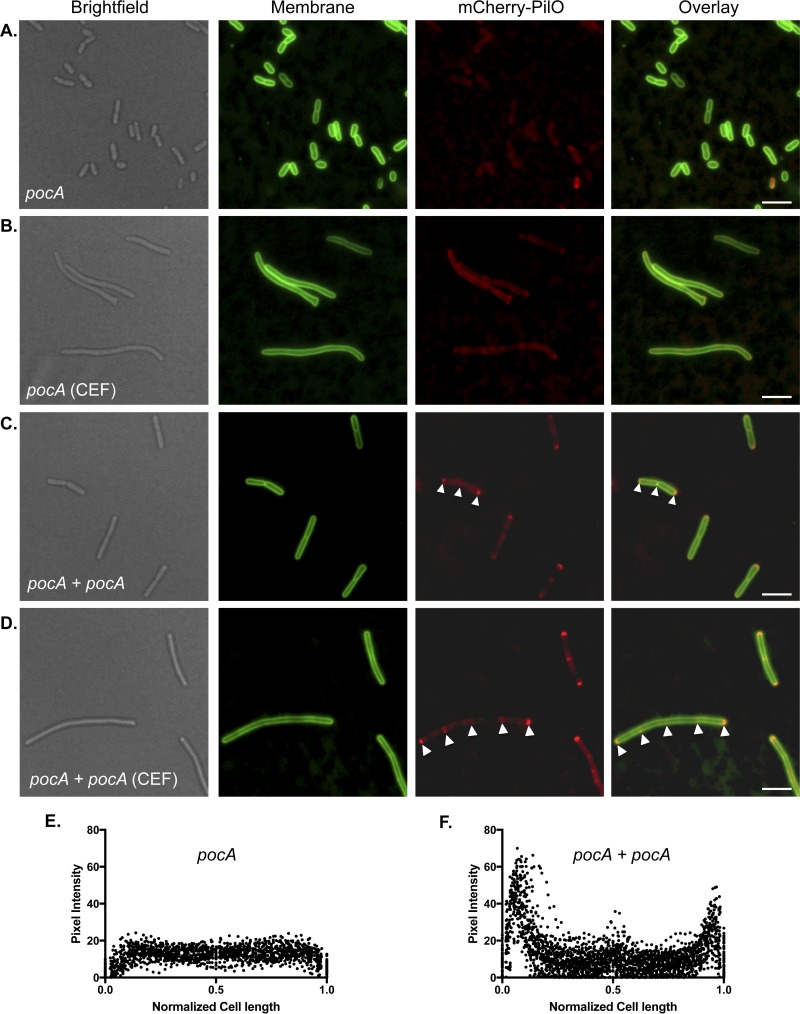
mCherry-PilO is delocalized in a *pocA* mutant. In the absence of polar organelle coordinating protein PocA, mCherry-PilO was delocalized in untreated cells (A) and cells treated with 40 μg/ml cefsulodin (CEF) (B). When *pocA* was reintroduced in *trans* (*pocA + pocA*), mCherry-PilO localization to the poles and septum was recovered (white arrowheads) in untreated cells (C), as well as to the poles and regularly spaced foci in CEF-treated cells (D). The last two panels show the quantification of mCherry pixel intensity in the absence of PocA (E) and in the *pocA*-complemented mutant (F) in 50 untreated cells after the normalization of length to 1 as described in Materials and Methods. Scale bars, 3 μm.

**FIG 8:**
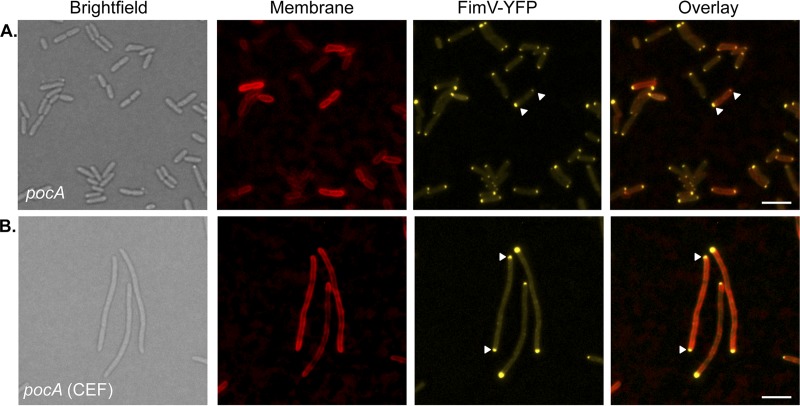
FimV remains polarly localized in the absence of PocA. (A) FimV-YFP remained localized to the cell poles (arrowheads) in a *pocA* mutant. (B) FimV-YFP localized to the poles in cefsulodin (CEF)-treated cells. Scale bars, 3 μm.

We focused on *pocA*, as the mutant’s ability to make a few pili suggested that the T4aP assembly system was functional, and deleted it from cells expressing either mCherry-PilO (Fig. 7A and B) or PilQ-mCherry (see Fig. S2E). mCherry-PilO was delocalized in the absence of *pocA*, and its WT localization was reestablished upon complementation with *pocA* in *trans* (Fig. 7C and D). PilQ-mCherry was delocalized in the *pocA* background, although some polar fluorescence remained (see Fig. S2E). Interestingly, while both PocA and FimV were required for T4aP assembly component localization to polar and midcell locations, they appear to act independently of one another. The fluorescent FimV fusion, which exhibits bipolar localization in WT cells, remained localized to the poles of a *pocA* mutant (Fig. 8).

## DISCUSSION

The T4aP machinery spans all of the layers of the Gram-negative cell envelope at the poles of rod-shaped cells, but how this megadalton protein complex is installed in the absence of dedicated PG-hydrolyzing enzymes was unclear. Here we showed that degradation of PG is likely unnecessary, because components of the *P. aeruginosa* T4P alignment subcomplex and the secretin are recruited to sites of future cell division, where they can be preinstalled, rather than retrofitted, into nascent poles. These data are consistent with our observation that mutants lacking multiple housekeeping PG hydrolases—which could potentially provide PG-lytic activities for retrofitting in the absence of system-specific enzymes—have correctly inserted, functional T4aP machines, as demonstrated by their ability to twitch (see Fig. S3). Because it is unlikely that we could delete all PG hydrolases without killing the cells, it remains possible that a housekeeping enzyme(s) could participate in T4aP complex insertion. However, the data presented here lead us to favor a different model, where at least three different mechanisms participate in the process of T4aP polar localization in *P. aeruginosa*. Two of these (PilQ and FimV) depend on the recognition of PG, as deletion of PilQ’s AMIN domains or FimV’s LysM motif results in delocalization.

10.1128/mBio.02103-16.4 FIG S3  Loss of multiple lytic transglycosylases (LTs) does not prevent twitching motility in *P. aeruginosa*. Twitching motility assays confirmed the functionality of the T4aP system in strains lacking all membrane-bound LTs (mLTs), soluble LTs (sLTs), family 1 LTs, and family 3 LTs (R. P. Lamers, U. T. Nguyen, Y. Nguyen, R. N. Buensuceso, and L. L. Burrows, Microbiologyopen **4:**879–895, 2015, doi:10.1002/mbo3.286; N. T. Blackburn and A. J. Clarke, J Mol Evol **52:**78–84, 2001). mLTs, MltA/B/D/F/F2; sLTs, SltB1/G/H/Slt; family 1 LTs, MltD/F/F2/Slt; family 3 LTs, SltB1/G/H/MltB. NP, nonpiliated. *n* = 3. Download FIG S3, PDF file, 0.1 MB. Copyright © 2017 Carter et al. 2017 Carter et al. This content is distributed under the terms of the Creative Commons Attribution 4.0 International license.

Cell poles are characterized by PG that has fewer stem peptides than lateral wall PG because of the activity of *N*-acetylmuramyl-l-alanine amidases that cleave the amide bond between *N*-acetylmuramic acid and stem peptides during daughter cell separation (31–33). This denuded form of PG is the binding target for multiple protein motifs, among them AMIN, SPOR, and LysM (28, 34–36). *P. aeruginosa* and *M. xanthus* PilQ monomers have N-terminal AMIN domains (two per monomer in *P. aeruginosa* and three in *M. xanthus*) that, in addition to targeting (37), may anchor secretin complexes to the PG layer to counter the substantial forces generated during twitching motility. *Neisseria gonorrhoeae* and *N. meningitidis* PilQ monomers also contain two AMIN domains at their amino termini (11), yet they express peritrichous T4aP. *Neisseria* spp. lack the cytoskeletal element MreB, which patterns the elongation of rod-shaped bacteria (38) and thus have a coccoid morphology. It is possible that the mechanism of T4aP assembly system recruitment to sites of cell division is similar in *Neisseria* spp., but because of their shape—the equivalent of two closely apposed poles—they appear to have peritrichous T4aP distribution.

In the *P. aeruginosa* T4aP system, at least two different PG-binding proteins were necessary for correct positioning. We showed previously (28) that FimV binds PG via its LysM motif, which is important for optimal secretin assembly. A number of recent studies have shown that FimV and related HubP proteins—which contain multiple protein-protein interaction domains in addition to conserved LysM motifs—act as landmarks that are responsible for the polar localization of other proteins, including those involved in the regulation of T4aP function, flagellar placement, and chromosome segregation (39–43). For example, *P. aeruginosa* FimL—a protein important for T4aP assembly via its regulation of intracellular cyclic AMP levels—interacts with the C-terminal TPR motif of FimV (39, 44). Loss of FimV also results in mislocalization of the core T4aP machinery (Fig. 6); thus, FimV appears to have both physical and regulatory roles in T4aP assembly and function.

Cowles et al. (29) reported that both the flagellum and T4aP were mislocalized in *P. aeruginosa* mutants lacking the polar organelle coordinating (Poc) proteins, but the mechanism remains enigmatic. The T4aP alignment subcomplex was delocalized in a *pocA* mutant (Fig. 7), while FimV remained bipolar (Fig. 8), implying that they act independently of one another—with the caveat that the FimV fusion used here was nonfunctional in twitching motility. PocAB-TonB3 are homologous to the ExbBD-TonB complex in *Escherichia coli* (29) which energizes siderophore uptake across the OM via direct interactions with the “Ton box” on one beta strand of TonB-dependent receptors. The N0 domains of PilQ monomers are structurally similar (2.3 Å root mean square deviation over 65 residues) to the signaling domain of FpvA, a TonB-dependent siderophore receptor in *P. aeruginosa* (45). This similarity hints at potential interactions between the TonB3 component of the Poc complex and the N0 domain of PilQ monomers. We noted that although deletion of *pocA* had no significant effect on cell morphology, complementation with a plasmid-borne copy of the gene increased the average cell length by ~50% (see Fig. S4), suggesting that the Poc system might influence the timing of cell division or affect PG remodeling. Subtle structural differences in PG architecture in the absence of PocA could impact the binding of proteins with PG-targeting motifs.

10.1128/mBio.02103-16.5 FIG S4  PocA overexpression increases cell length. *pocA* mCherry*-pilO* was transformed with the empty vector (pB) or a PocA expression construct (pB-*pocA*). As controls, WT PAK and mCherry-*pilO* were also examined. Transformed cells were grown overnight and stab inoculated into a chambered 1.0 borosilicate cover glass (LabTek) containing LB with 1.0% (wt/vol) agar. Slides were incubated for 1.5 h at 37°C. (A) Cells were visualized with an EVOS FL Auto microscope with an attached EVOS Onstage Incubator set to 37°C and a 63× oil immersion objective. Scale bar, 5 μm. (B) Cell length analysis was performed with the MicrobeJ plugin (A. Ducret, E. M. Quardokus, and Y. V. Brun, Nat Microbiol **1:**16077, 2016) for ImageJ (C. A. Schneider, W. S. Rasband, and K. W. Eliceiri, Nat Methods **9:**671–675, 2012) as described in Text S1. The numbers of cells analyzed were as follows: PAK, 601; mCherry, 651; *pocA* mCherry*-pilO* + pB, 602; *pocA* mCherry*-pilO* + pB-*pocA*, 558. Download FIG S4, TIF file, 0.6 MB. Copyright © 2017 Carter et al. 2017 Carter et al. This content is distributed under the terms of the Creative Commons Attribution 4.0 International license.

Midcell recruitment of mCherry-PilO was dependent on the secretin monomer PilQ (Fig. 3C and D), but deletion of the pilotin PilF had no effect (Fig. 5), showing that recruitment and confinement of PilMNOPQ to future cell division sites occur prior to secretin oligomerization. Prior studies of *M. xanthus* T4aP assembly system formation suggested that PilQ multimerization is essential for the recruitment of additional T4aP assembly components because loss of Tgl—the *M. xanthus* PilF homolog—abrogated the polar localization of PilQ and other T4aP proteins. These data supported an “outside-in” model of *M. xanthus* T4aP system formation, dependent on PilQ oligomerization (26). Interestingly, *M. xanthus* PilQ was suggested in that study to localize to the septum either “late during cell division or immediately after.” These data are consistent with the pattern of PilQ localization in *P. aeruginosa*; however, the pilotin proteins in *M. xanthus* and *P. aeruginosa* appear to play somewhat different roles.

Together, the data led us to propose a pathway for T4aP assembly machinery integration into the *P. aeruginosa* cell envelope in the absence of dedicated PG hydrolases. We suggest that PilMNOPQ are cotranslated, forming an IM-bound subcomplex that diffuses laterally until the AMIN domains of PilQ monomers bind to septal PG. FimV, which has the capacity to interact with septal PG via its Lys motif, may also interact with components of the subcomplex to retain them at cell division sites. The next steps are still unclear, as the oligomerization pathway for PilQ in the OM remains unknown. We previously proposed (9) that cotranslocation of the lipoprotein PilF and PilQ monomers by the Lol system might promote PilQ insertion into the OM, where it oligomerizes. In this new model, we suggest that PilF is translocated independently by the Lol system to the OM, where it then scans for, binds to, and promotes the translocation of PilQ monomers from the IM to the OM during invagination in the final stages of cell division.

Two lines of evidence support this new hypothesis. First, in *M. xanthus*, coincubation of *pilQ* and *tgl* (*pilF*) mutants, neither of which is able to twitch, allowed for intercellular transfer of Tgl from the *pilQ* mutant to the *tgl* mutant and oligomerization of the latter’s PilQ monomers, restoring piliation and motility (46). Thus, Tgl already present in the OM of the *pilQ* mutant could promote the insertion and oligomerization of PilQ monomers in the *tgl* mutant. Second, the ability of OM lipoproteins to interact with IM proteins across the periplasm is now well established, as exemplified by *E. coli* OM lipoproteins LpoA and LpoB (47, 48). These proteins are essential for stimulating the activities of the IM PG synthases PBP1a and PBP1b, respectively, through direct interactions with regulatory domains of those enzymes. Once PilQ monomers reach the OM with the assistance of PilF, they may first form conformational intermediates prior to maturation into stable, SDS-resistant oligomers. In *N. gonorrhoeae*, certain point mutations in the secretin domain were associated with the formation of immature, leaky oligomers, rendering the cells more susceptible to beta-lactam antibiotics (49).

The above scenario for PilQ translocation suggests that the ~2-nm porosity of PG (50) would not pose a physical barrier to secretin assembly, as individual monomers (~77 kDa) are small enough to pass through its natural gaps. Retention of PilQ AMIN interactions with septal PG during the translocation and insertion of the C-terminal secretin domain in the OM would ultimately place the AMIN domains “under” the PG layer, where they would be ideally positioned to counteract retraction forces imposed during twitching motility. Because polar PG is less likely to be remodeled because of its limited side chain content, the secretin would remain stably fixed to the cell wall. Studies of the half-life of a single bacterial cell pole show that it can be hundreds of generations old (51); thus, it is perhaps not surprising that secretin complexes are highly resistant to denaturation, allowing them to last as long as the poles in which they are embedded.

Among the questions remaining is whether the interaction interface between PilP and PilQ is altered during the translocation of PilQ monomers to the OM. We established that midcell localization of mCherry-PilO depends on PilP (Fig. 3F), which interacts with the N0 domain of PilQ monomers via its C-terminal HR domain (11, 20). PilP also interacts with PilNO heterodimers via its long unstructured N terminus (17). This flexible tether might allow the C-terminal domain of PilP to remain connected to the N0 domain of PilQ as secretin monomers transit from the IM to the OM. Alternatively, PilP may be repositioned relative to PilQ when the latter translocates to the OM. In the evolutionarily related T2SS, multiple interaction interfaces between the HR domain of GspC (PilP) and N0 of GspD (PilQ) have been identified through a combination of structural, biochemical, and functional studies (45, 52, 53). These various interfaces—some of which are mutually exclusive—were attributed to experimental setup differences but could also reflect the capture of different interaction states before and after secretin oligomerization. Studies to address these possibilities are under way.

## MATERIALS AND METHODS

### Strains, media, and growth conditions.

The bacterial strains, plasmids, and primers used in this study are listed in Tables 1 and 2. *E. coli* and *P. aeruginosa* were grown at 37°C in LB (Lennox broth) or on LB 1.5% agar plates supplemented with antibiotics. The antibiotics and their respective concentrations were ampicillin (Ap) at 100 μg/ml, carbenicillin (Cb) at 200 μg/ml, and gentamicin (Gm) at 15 μg/ml for *E. coli* strains and 30 μg/ml for *P. aeruginosa* strains, unless otherwise stated. Plasmids were transformed by heat shock into chemically competent *E. coli* cells or by electroporation into *P. aeruginosa* suspended in sterile water. All constructs were verified by DNA sequencing (MOBIX Lab, McMaster University).

### Generation of *P. aeruginosa* mutants.

Mutants were made by previously described methods (54). Deletion constructs for the generation of *minC* and *pilF* mutants were designed to include 500 nucleotides up- and downstream of the gene to be deleted, as well as the first and last 50 nucleotides of the gene. Constructs were synthesized and cloned into pUC57 (GenScript, Piscataway, NJ). Deletion inserts in pUC57 were subcloned into the pEX18Gm suicide vector (55) at the 5′ BamHI and 3′ HindIII restriction sites and verified by DNA sequencing. After verification, suicide vectors containing the mutant gene (*pilF* or *minC*) were transformed into *E. coli* SM10 cells. Plasmids were then transferred by conjugation at a 1:9 ratio of *P. aeruginosa* to *E. coli*. The mixed culture was pelleted for 1 min at 2,292 × *g*, and the pellet was resuspended in a 100-μl aliquot of LB, spot plated onto LB agar, and incubated overnight at 37°C. The mating mixture was removed from the LB agar with a sterile toothpick and resuspended in 1 ml of LB, and the *E. coli* SM10 donor was counterselected by plating the mixture on *Pseudomonas* isolation agar (PIA; Difco) containing Gm (100 μg/ml). Gm-resistant *P. aeruginosa* colonies were streaked onto LB no-salt plates containing sucrose (1% [wt/vol] Bacto tryptone, 5% [wt/vol] sucrose, 1.5% agar, 0.5% [wt/vol] Bacto yeast extract) and incubated for 16 h at 30°C. Selected colonies were then subcultured on LB agar and on LB agar plus Gm, and Gm-sensitive colonies were screened by PCR with appropriate primers (Table 2) to confirm integration. Amplicons from colonies with the desired PCR profile were sequenced to confirm the incorporation of the mutation, and the *pilF* mutant was subjected to Western blot analysis with an anti-PilF antibody to verify loss of the gene product.

A *pilQ* knockout construct containing an FRT-flanked Gm^r^ cassette was designed previously (9). This construct was integrated into the PAK chromosome with a Flp-FRT recombination system (55) and selected for using the methods described above, with the following modifications. Following growth on sucrose, colonies were cultured on LB agar, LB agar with Gm, and LB agar plus Cb. Colonies that had undergone double recombination, integrating *pilQ*::GmFRT into the chromosome without pEX18Ap (Cb-sensitive colonies), were selected. The Gm^r^ cassette was removed by Flp recombinase-catalyzed excision by conjugally transferring Flp-expressing pFLP2 from SM10 cells into PAK cells carrying the GmFRT-disrupted *pilQ* gene. SM10 cells were counterselected on PIA containing Cb (200 μg/ml). pFLP2 was removed from *P. aeruginosa* by streaking Cb-resistant colonies onto LB agar with no salt containing 5% (wt/vol) sucrose for 16 h at 30°C. Colonies were then cultured on LB agar and on LB agar containing Gm or Cb. Cb- and Gm-sensitive colonies were screened by PCR with the PilQ FRT-Chk primers listed in Table 2 to confirm the retention of the FRT scar. The *pilQ* genes of mutant strains were sequenced to confirm correct incorporation of the mutation and analyzed by Western blotting with anti-PilQ serum to confirm loss of the gene product.

The *pocA* deletion construct was created previously in the pEX18Tc suicide vector (29). The *pocA* deletion insert was subcloned into pEX18Gm with EcoRI and XbaI, and constructs were verified by DNA sequencing. After verification, the suicide vector containing the *pocA* deletion construct was transformed into *E. coli* SM10 cells and the mutation was introduced into *P. aeruginosa* as described above.

### Generation of a chromosomally encoded mCherry-PilO fusion.

A construct encoding a fusion of mCherry (56) to the N terminus of PilO, plus 700 nucleotides upstream of *pilO* and 700 nucleotides downstream of *pilO*, was synthesized and cloned into pUC57 (GenScript, Piscataway, NJ). The mCherry-PilO-encoding insert was subcloned into suicide vector pEX18Gm at the 5′ EcoRI and 3′ HindIII restriction sites, and constructs were verified by DNA sequencing and introduced into chemically competent *E. coli* SM10 by heat shock. The plasmid was then transferred by conjugation at a 1:9 ratio of *P. aeruginosa* (WT or various T4aP mutants) to *E. coli* as described above. Following counterselection and curing of merodiploids by sucrose selection, integration of the mCherry-PilO fusion into Gm-sensitive colonies was confirmed by PCR and DNA sequencing (Table 2). mCherry-PilO-expressing strains were analyzed by Western blot analysis and twitching assays to verify the expression of an intact fluorescent fusion protein and—in the WT—the function of the T4aP machinery, respectively.

### Generation of complementation constructs.

The *pilQ*, *pocA*, and *pilF* genes were amplified by PCR with PAK chromosomal DNA as the template (Table 2) and cloned into pBADGr (Table 1). The digested DNA was purified and ligated into pBADGr with T4 DNA ligase in accordance with the manufacturer’s instructions. Ligation mixtures were then transformed into *E. coli* DH5α cells and grown overnight at 37°C on LB agar supplemented with the appropriate antibiotics.

**TABLE 1:** Bacterial strains and plasmids used in this study

Strain or plasmid	Description	Source
*E. coli* strains		
DH5α	F^−^ φ80d*lacZ*ΔM15 Δ(*lacZYA-argF*)*U169 recA1 endA1 hsdR17*(r_K_^−^ m_K_^−^) *phoA supE44 thi-1 gyrA96 relA1* λ^−^	Invitrogen
SM10	*thi-1 thr leu tonA lacy supE recA* RP4-2-Tcr::Mu, Km^r^; mobilizes plasmids into *P. aeruginosa* via conjugation	62
*P. aeruginosa* strains		
PAK	WT	J. Boyd
Δ*pilM*	Deletion of *pilM*	63
*pilP*::FRT	FRT scar at nucleotide 86 of *pilP*	14
*pilQ*::FRT	FRT scar at nucleotide 571 of *pilQ*	9
*pilA*::FRT	FRT scar at SphI site within *pilA*	64
Δ*pilF*	*pilF* deletion strain	This study
Δ*minC*	*minC* deletion strain	This study
mCherry-PilO	In-frame fusion of mCherry to 5′ end of *pilO* on PAK chromosome	This study
Δ*pocA*	*pocA* deletion strain	29
Δ*fimV*	*fimV* deletion strain	39
*fimV*Δ*LysM*	*fimV* with in-frame deletion of LysM domain	28
mCherry-PilO Δ*pilM*	Deletion of *pilM* in mCherry-PilO strain	This study
mCherry-PilO *pilP*::FRT	FRT scar in *pilP* at nucleotide 86 in mCherry-PilO strain	This study
mCherry-PilO *pilQ*::FRT	FRT scar at position 571 within *pilQ* in mCherry-PilO strain	This study
mCherry-PilO Δ*pilF*	Deletion of *pilF* in mCherry-PilO strain	This study
mCherry-PilO Δ*minC*	Deletion of *minC* in mCherry-PilO strain	This study
mCherry-PilO Δ*pocA*	Deletion of *pocA* in mCherry-PilO background	This study
mCherry-PilO Δ*fimV*	Deletion of *fimV* in mCherry-PilO background	This study
mCherry-PilO *fimV*Δ*LysM*	Insertion of mCherry at 5′ end of *pilO* with *fimV* containing deletion of LysM domain	This study
Plasmids		
pEX18Gm	Suicide vector used for gene replacement, Gmr^r^	55
pEX18Ap	Suicide vector used for gene replacement, Ap^r^	55
pBADGr	Arabinose-inducible expression vector	58
pFLP2	Suicide vector containing Flp recombinase	55
pEX18Gm::mCherry*-pilO*	Suicide vector containing mCherry*-pilO*, subcloned from pUC57 (GenScript) with EcoRI/HindIII	This study
pEX18Gm::Δ*pilF*	Suicide vector containing *pilF* deletion, cloned with BamHI/HindIII	This study
pEX18Gm::Δ*minC*	Suicide vector containing *minC* deletion, cloned with BamHI/HindIII	This study
pEX18Gm::Δ*pocA*	Suicide vector containing *pocA* deletion, cloned with EcoRI/XbaI	This study
pEX18Ap-*pilQ*::GmFRT	Suicide vector containing PAK *pilQ* disrupted with FRT-flanked Gm cassette at position 571	This study
pBADGr::*pilQ-*mCherry	*pilQ-*mCherry complementation construct, cloned with EcoRI/HindIII	This study
pBADGr::*pilF*	*pilF* complementation construct, cloned with SphI/HindIII	This study
pBADGr::*pilQ*	*pilQ* complementation construct, cloned with EcoRI/HindIII	This study
pBADGr::*pilQ*Δ*AMIN*	*pilQ*Δ*AMIN* complementation construct, cloned with EcoRI/HindIII	This study
pBADGr::*pocA*	*pocA* complementation construct, cloned with EcoRI/HindIII	This study
pBADGr::*fimV-yfp*	*fimV-yfp* complementation construct, cloned with KpnI/XbaI	This study

A construct encoding a truncated version of PilQ lacking its AMIN domains was previously generated in the PAO1 strain (9). The same boundaries were used here to generate a truncated version of PAK PilQ. The PilQ Clone Fwd and PilQ 5′ Rev primers were used to amplify the 5′ end of the gene, excluding the first AMIN domain, and the PilQ N0 Fwd and PilQ Clone Rev primers (Table 2) were used to amplify the remainder of the gene, beginning at the N0 domain and excluding the second AMIN domain. Purified amplicons were digested with XbaI and ligated into pBADGr with T4 DNA ligase. The ligation product and pBADGr were then digested with EcoRI and HindIII and ligated by the same protocol. The fidelity of the pBADGr-*pilQ* construct was confirmed by sequencing with the pBADGr mcs Fwd and Rev primers (Table 2).

**TABLE 2:** Sequences of primers used in this study

Primer name	Oligonucleotide sequence
PilF-Fwd	5′ACGCCTTGCAAGATCAACCTGATTCCG3′
PilF-Rev	5′TCGAACGCGCCGTTTTCCAGCTGACGC3′
PilF mid-Rev	5′GGCCGGTTTCTTCATTTGCAGCG3′
PilF Clone-Fwd	5′TCATGCATGCATGACTGTACGCGCCGCGCTGG3′
PilF Clone-rev	5′TCATAAGCTTTCATTTTTCCGCCTGGAATTCCTG3′
MinC Fwd	5′GGATACGGCAACTGCACCACCAGCCAG3′
MinC Rev	5′GACGACGTTGACGAAGTCGTACACCAC3′
MinC mid-Rev	5′CGTGGCGGCGACAGACCTCGAGGA3′
PilN 3′-Fwd	5′GCCAACGTGTTCCAACTG3′
PilO Rev	5′CCACGCTGATCTGGATCG3′
PocA Fwd	5′GAATTCGGGGATATGCCACGTGTGGGA3′
PocA Rev	5′AAGCTTTGCGGCGGAATTTCACGCTTTGC3′
PocA mid-rev	5′GAGGATCTCTCCCAGCGG3′
PocA Clone-Fwd	5′TCATGAATTCGTGTGGGAACTGGTTCAAGCCGG3′
PocA Clone-rev	5′TCATAAGCTTTCACGCTTTGCCTTCCTCGACGTAG3′
PilQ Clone-Fwd	5′AGAATTCCAACAGCAGTCTGTACAA3′
PilQ Clone-rev	5′TCATAAGCTTTCAGCGACCGATTGCGATGGCCTG3′
PilQ 910-Fwd	5′CGACCTGAATCTGGTGGC3′
PilQ FRT-Chk Fwd	5′TTGATCATCAACCTGACCGCGCTGTCG3′
PilQ FRT-Chk rev	5′TCATCGGCTTGATGCTGACGGTCAGG3′
pBAD mcs-Fwd	5′AAGTGTCTATAATCACGGCAGA3′
pBAD mcs-Rev	5′TCACTTCTGAGTTCGGCATGG3′
mCherry Rev	5′TCATGCATGCGCTTCCGCCGCTCTTGTACAGCTCGTCCATGCCGC3′
PilQ 5′ Rev	5′TCATTCTAGAGTCCGCGGCGAGCAATGCCGGCGC3′
PilQ N0 Fwd	5′TCATTCTAGAGGCGAGAAACTGTCGCTGAACTTC3′
FimV Clone Fwd	5′GCGGGTACCATGGTTCGGCTTCG3′
FimV Clone rev	5′GCGTCTAGAGGCCAGGCGCTCCA3′

Since PilQ has a cleavable N-terminal signal sequence and a C terminus that is exposed to the extracellular environment (9, 57), we designed a construct encoding an internal fusion of mCherry to PilQ (between the first and second AMIN domains, residues 132 and 133), which was synthesized and subcloned into pUC57 (GenScript). The PilQ-mCherry-encoding insert was subcloned into complementation vector pBADGr (58) at the 5′ EcoRI and 3′ HindIII restriction sites and verified by DNA sequencing. The construct was then introduced into PAK *pilQ*::FRT cells by electroporation. Expression of the PilQ-mCherry fusion was verified by Western blot analysis with anti-PilQ and anti-mCherry antibodies, and function was verified with twitching assays (59).

The *yfp* gene, which encodes yellow fluorescent protein (YFP), was cloned into the HindIII restriction site of pBADGr. Correct insertion of the pBADGr-*yfp* construct was confirmed by DNA sequencing with mcs Fwd/Rev primers (Table 2). A version of *fimV* lacking its stop codon was amplified with the FimV Clone Fwd/Rev primers (Table 2) and cloned into pBADGr in frame with the *yfp* gene by digestion of both the vector and the insert with restriction enzymes KpnI and XbaI and ligation with T4 DNA ligase. Successful ligation of the insert was confirmed by using the pBADGr multiple cloning site flanking primers (Table 2) and DNA sequencing. The construct was then electroporated into Δ*fimV* and Δ*pocA* mutant cells. Stable fusion of YFP to the C terminus of FimV was confirmed by Western blotting with anti-AFP and anti-FimV antibodies. This fusion is considered nonfunctional, as it failed to restore twitching in the *fimV* background.

### Twitching motility assays.

Twitching motility assays were performed as previously described (59). Single colonies were stab inoculated into the bottom of a 1% LB agar plate. Plates were incubated for 24 h at 30°C. Following incubation, the agar was removed, and the adherent bacteria were stained with 1% (wt/vol) crystal violet for 30 min and then washed with water to remove the unbound dye. Twitching zones were photographed, and their areas were measured with Fiji (60). All experiments were performed in triplicate with at least three independent replicates.

### Analysis of whole-cell lysates by Western blotting.

Cultures were grown overnight at 37°C in LB supplemented with appropriate antibiotics and diluted to an OD_600_ of 0.6. A 1-ml aliquot of cells was collected by centrifugation at 2,292 × *g* for 1 min, and the cell pellet was resuspended in 100 μl of SDS sample buffer (80 mM Tris [pH 6.8], 5.3% [vol/vol] 2-mercaptoethanol, 10% [vol/vol] glycerol, 0.02% [wt/vol] bromophenol blue, 2% [wt/vol] SDS) and boiled for 10 min.

Equal volumes of whole-cell lysates were separated by 12.5% SDS-PAGE at 80 to 150 V and transferred to nitrocellulose membranes for 1 h at 225 mA. Membranes were blocked with 5% (wt/vol) low-fat skim milk powder in a phosphate-buffered saline (PBS) solution at pH 7.4 for 1 h at room temperature and then incubated with the appropriate polyclonal (for PilF, PilM, PilO, PilP, PilQ, and FimV) or monoclonal (for mCherry) antiserum for 1 h at room temperature. Membranes were washed twice in 10 ml of PBS for 5 min and then incubated in either a goat anti-rabbit or a goat anti-mouse IgG alkaline phosphatase-conjugated secondary antibody (Bio-Rad) at a dilution of 1:3,000 for 1 h at room temperature. Membranes were washed twice in PBS for 5 min and developed in a solution containing 100 μl of nitroblue tetrazolium and 100 μl of 5-bromo-4-chloro-3-indolylphosphate (BCIP) in 10 ml of alkaline phosphatase buffer (100 mM NaCl, 5 mM MgCl_2_, 100 mM Tris, pH 9.5).

### Cell preparation for transmission electron microscopy.

Cells from a 1-ml overnight culture in LB were pelleted at 2,292 × *g* and resuspended in 1 ml of a solution of 2% (wt/vol) glutaraldehyde in 0.1 M sodium cacodylate buffer at a pH of 7.4. Cells were fixed in solution for 2 h at 4°C and washed twice with cold sodium cacodylate buffer (pH 7.4). Samples were then treated with 1% osmium tetroxide in 0.1 M sodium cacodylate for 1 h and stained with 2% uranyl acetate overnight. After staining, cells were dehydrated gradually with ethanol, treated with propylene oxide, embedded in Spurr’s resin, and polymerized at 60°C overnight. The polymerized samples were sectioned with a Leica UCT ultramicrotome and poststained with 2% uranyl acetate and lead citrate. The prepared specimens were examined in McMaster University’s Electron Microscopy Facility with a JEOL JEM 1200 EX TEMSCAN microscope (JEOL, Peabody, MA) operating at an accelerating voltage of 80 kV. Images were acquired with an AMT 4-megapixel digital camera (Advanced Microscopy Techniques, Woburn, MA).

### Fluorescence microscopy.

Strains were incubated overnight at 37°C in 5 ml of LB supplemented with the appropriate antibiotics. One milliliter of cells was pelleted at 2,292 × *g* and resuspended in 1 ml of sterile water containing 10 μg/ml Fm1-43 Fx (for cells expressing mCherry) or Fm1-64 Fx (for cells expressing YFP) membrane stain (Invitrogen). Cells were mixed with dye by gentle pipetting and pelleted at 2,292 × *g*. Pelleted cells were then stab inoculated with a pipette tip into the coverslip-medium interface at the bottom of an eight-well microscope glass coverslip slide containing 200 μl of 1% LB agarose per well (Lab-Tek). Cells were then incubated for 75 min at 37°C prior to imaging. In filamentation experiments, 1 ml of cells grown overnight was subcultured in 4 ml of LB containing 40 μg/ml cefsulodin and incubated for 3 h at 37°C. Cells were then stained and mounted for microscopy by the above-described protocol. Cells were imaged with an EVOS FL Auto microscope in the McMaster Biophotonics Facility. Images were acquired with a 60× oil immersion objective with a Texas Red filter, a YFP filter, or transmitted white light (no filter). Fiji (60) was used to adjust image brightness and contrast and to overlay bright-field and fluorescence images.

### Quantification of mCherry-PilO fluorescence.

Fluorescence was quantified with Fiji software  (60). Each field in the Texas Red (for mCherry) and YFP (for Fm1-43 Fx) filter sets was overlaid and used with the micrograph from the YFP filter for image analysis. A 250-μm^2^ grid was added to each field for precise documentation of the quantified cells. Moving systematically between squares in the grid, cells that fulfilled the following criteria were selected: (i) not contacting other cells, (ii) normal rod morphology, and (iii) a stable mCherry-PilO fusion (diffuse cytoplasmic fluorescence implies cleavage of the tag in that cell). Cell lengths and pixel intensities were measured from one cell pole to the other and normalized to 1 (i.e., each data point associated with length divided by the total length of the cell to yield a normalized 0-to-1 scale). Pixel intensity measurements in the YFP field (cell outline) were then subtracted from the overlay field, producing mCherry-PilO pixel intensity measurements alone in each cell on a scale of 0 to 1. From the data, an *x*-*y* scatter plot was generated to show the distribution of mCherry-PilO fluorescence over cell length.
