# Supplementary material for: The Type IVa Pilus Machinery Is Recruited to Sites of Future Cell Division
Source: mBio. 2017 Jan 31;8(1):e02103-16. doi: 10.1128/mBio.02103-16 (PMC5285504; doi:10.1128/mBio.02103-16)
Supplement: TEXT S1 [file mbo001173172s1.docx]

**Supplementary data for Carter et al.**

**SUPPLEMENTARY METHODS**

*Peptidoglycan purification*

Peptidoglycan was purified as described in Wehbi et al. 2011. Four hundred mL of mPAO1 was grown to OD_600_ of 0.8. After cooling, cells were harvested at 6,000 x g for 15 min at 4^o^C. The pellet was resuspended in 6 ml ice cold water and then added dropwise into 6 ml of boiling 8% SDS. Samples were boiled for 30 min and then cooled and diluted to 25 ml with water. PG was harvested via centrifugation for 30 min at 46,000 x g and 4**°**C, and the pelleted PG was washed 2x with deionized water. After washing, the PG pellet was harvested as above and resuspended in 10 ml of 10 mM tris pH 8.0, 10 mM NaCl, 0.34 M imidazole and 20 mM MgSO4 and sonicated 1 min on a Misonix sonicator with microtip. To that, 100 μg/ml of α-amylase, 10 μg/ml DNase, 50 μg/ml RNase were added, and the mixture incubated for 2 h at 37**°**C. Pronase was added at 200 μg/ml and incubated at 60**°**C for 2 h. Enzymatically treated PG was then added dropwise to 10 mL 8% SDS at 80**°**C. After cooling, the PG was harvested and washed as above, resuspended in water, and lyophilized.

*PilQ fragment purification*

PilQ fragments were purified as described in Tammam et al. {Tammam, 2013 #7}. PilQ_24-445_ includes the entire periplasmic domain, PilQ_24-280_ includes the AMIN domains, and PilQ_281-445_ includes the N0 and N1 domains. After purification on a NiNTA column samples were dialyzed overnight at 4**°**C into 20 mM tris pH 7.5, 150 mM NaCl.

*Peptidoglycan pull-down experiment*

Pull-down experiments were performed as described in Wehbi et al. {Wehbi, 2011 #6}. Purified PG 625 mg was mixed with 50 mg of purified PilQ fragments. The samples were then diluted to 500 ml with buffer (20 mM tris pH 7.5, 150 mM NaCl). A control reaction without PG was set up for each PilQ fragment. After incubation at 4**°**C with rocking for 2 h, samples were centrifuged at 21,000 x g at 4**°**C in a bench top microfuge. The supernatant of the control reaction is the initial fraction while the supernatant of the +PG sample is the unbound fraction. After removing the supernatant from the +PG sample, the pellet was washed with 500 mL of 50 mM tris and centrifuged as above. The supernatant of this spin is the wash fraction. Finally, bound proteins were eluted with 500 mL 50 mM tris and 4% SDS (incubated 15 min at room temperature with rocking) and centrifuged as above. The supernatant of this third spin is the bound fraction. Samples were loaded on a gel and blotted with an anti-6His antibody conjugated to alkaline phosphatase and developed with NBT-BCIP. A protein control reaction containing 50 mg of BSA was also performed and analyzed by Coomassie stained acrylamide gel, and BSA did not bind to PG (data not shown).

*Cell length analysis for pocA strains*

Quantification of cell length was performed using the MicrobeJ plugin {Ducret, 2016 #109} for ImageJ {Schneider, 2012 #110}. Brightfield micrographs were processed on ImageJ using the ‘subtract background’ command. Processed images were loaded into MicrobeJ, and bacteria ROIs were fit to rod-shaped and thresholded as follows: area 0.4μm^2^-max; length: 0.3μm-max; width 0.2μm-1.5μm. Pixel intensity profile plots for each cell were also generated using the following criteria (thickness 1 μm; extension 0.5μm). In cases where the software detected incomplete cells, or two cells were detected as one particle, these particles were removed manually from the analysis. Data are representative of 3 separate trials. Cell length data was exported to GraphPad Prism 5 (GraphPad Software) and plotted as frequency distribution, and a nonlinear regression line was generated using Gaussian distribution.

**SUPPLEMENTARY REFERENCES**

1. Lamers RP, Nguyen UT, Nguyen Y, Buensuceso RN, Burrows LL. 2015. Loss of membrane-bound lytic transglycosylases increases outer membrane permeability and beta-lactam sensitivity in Pseudomonas aeruginosa. MicrobiologyOpen. 4(6):879-95. doi: 10.1002/mbo3.286.

2. Blackburn NT, Clarke AJ. 2001. Identification of four families of peptidoglycan lytic transglycosylases. J Mol Evol 52:78-84.

3. Ducret A, Quardokus EM, Brun YV. 2016. MicrobeJ, a tool for high throughput bacterial cell detection and quantitative analysis. Nat Microbiol 1:16077.

4. Schneider CA, Rasband WS, Eliceiri KW. 2012. NIH Image to ImageJ: 25 years of image analysis. Nature methods 9:671-675.
