# Supplementary figures and images for: The Type IVa Pilus Machinery Is Recruited to Sites of Future Cell Division
Source: mBio. 2017 Jan 31;8(1):e02103-16. doi: 10.1128/mBio.02103-16 (PMC5285504; doi:10.1128/mBio.02103-16)

Carter et al. Figure S1

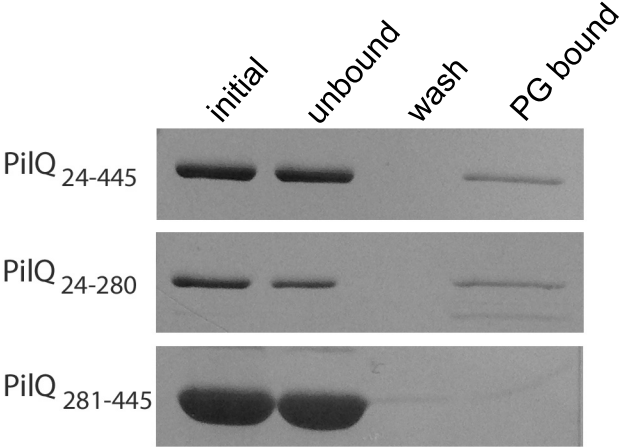

Supplement: FIG S1 [file mbo001173172sf1.pdf]

Carter et al., Fig. S2

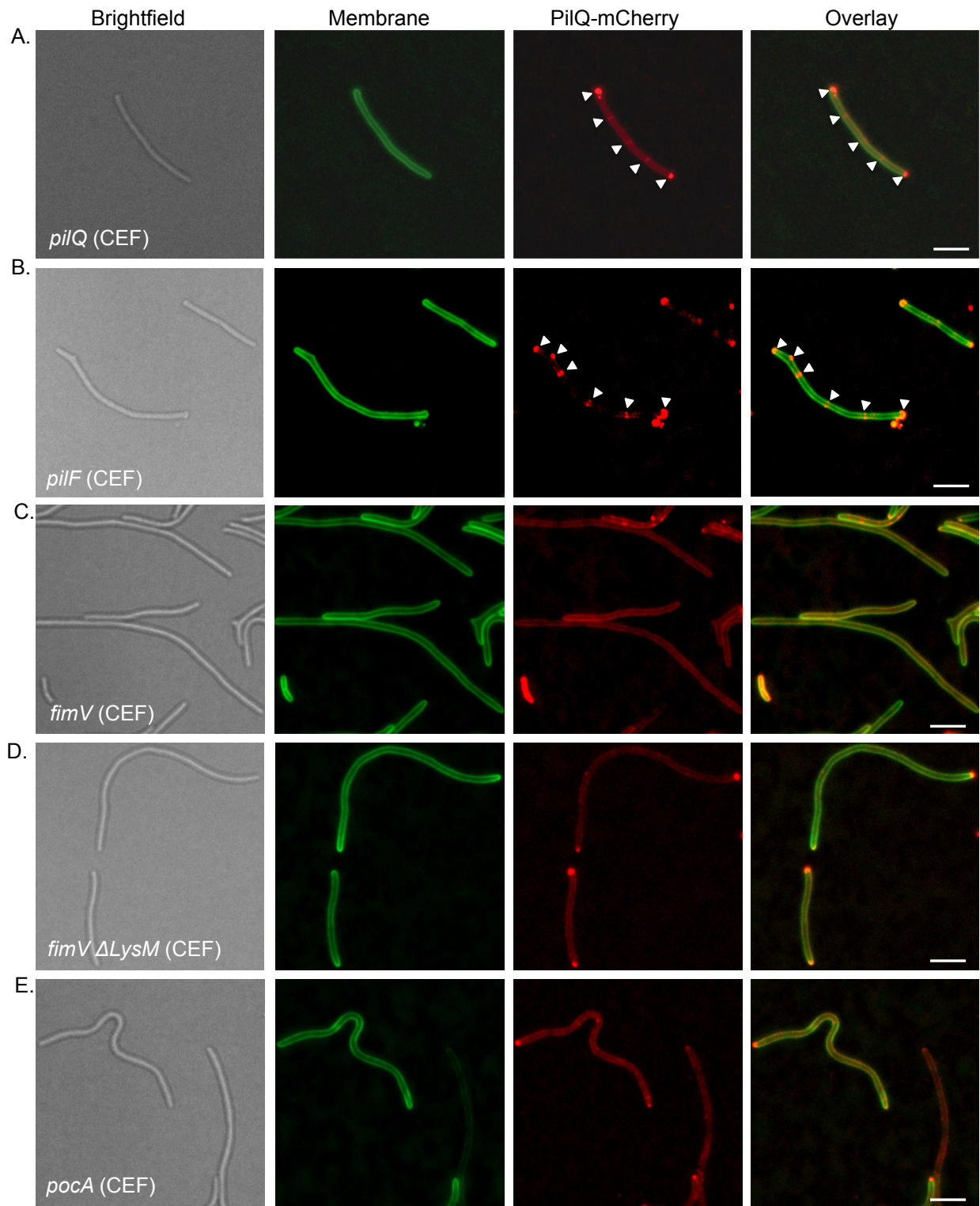

Supplement: FIG S2 [file mbo001173172sf2.pdf]

Carter et al. Figure S3

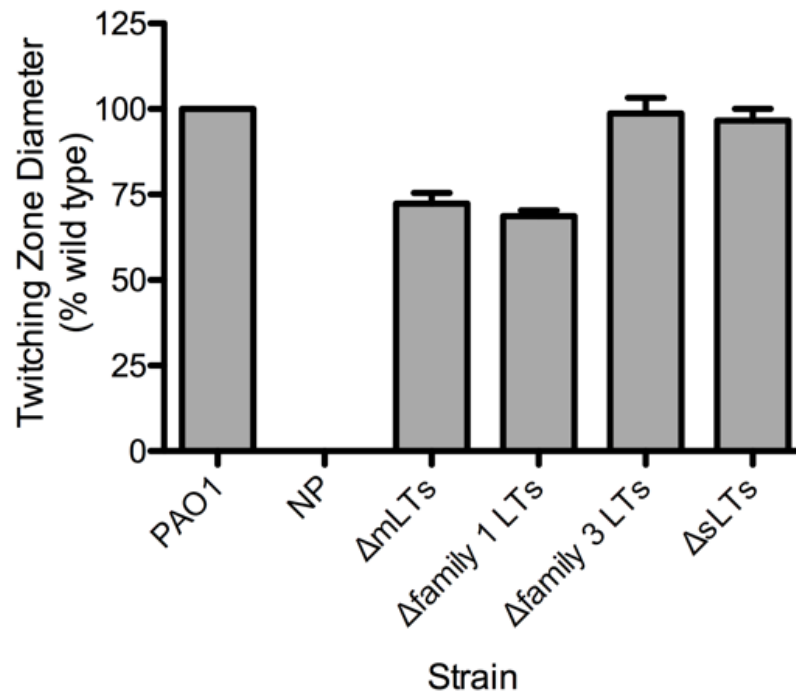

Supplement: FIG S3 [file mbo001173172sf3.pdf]

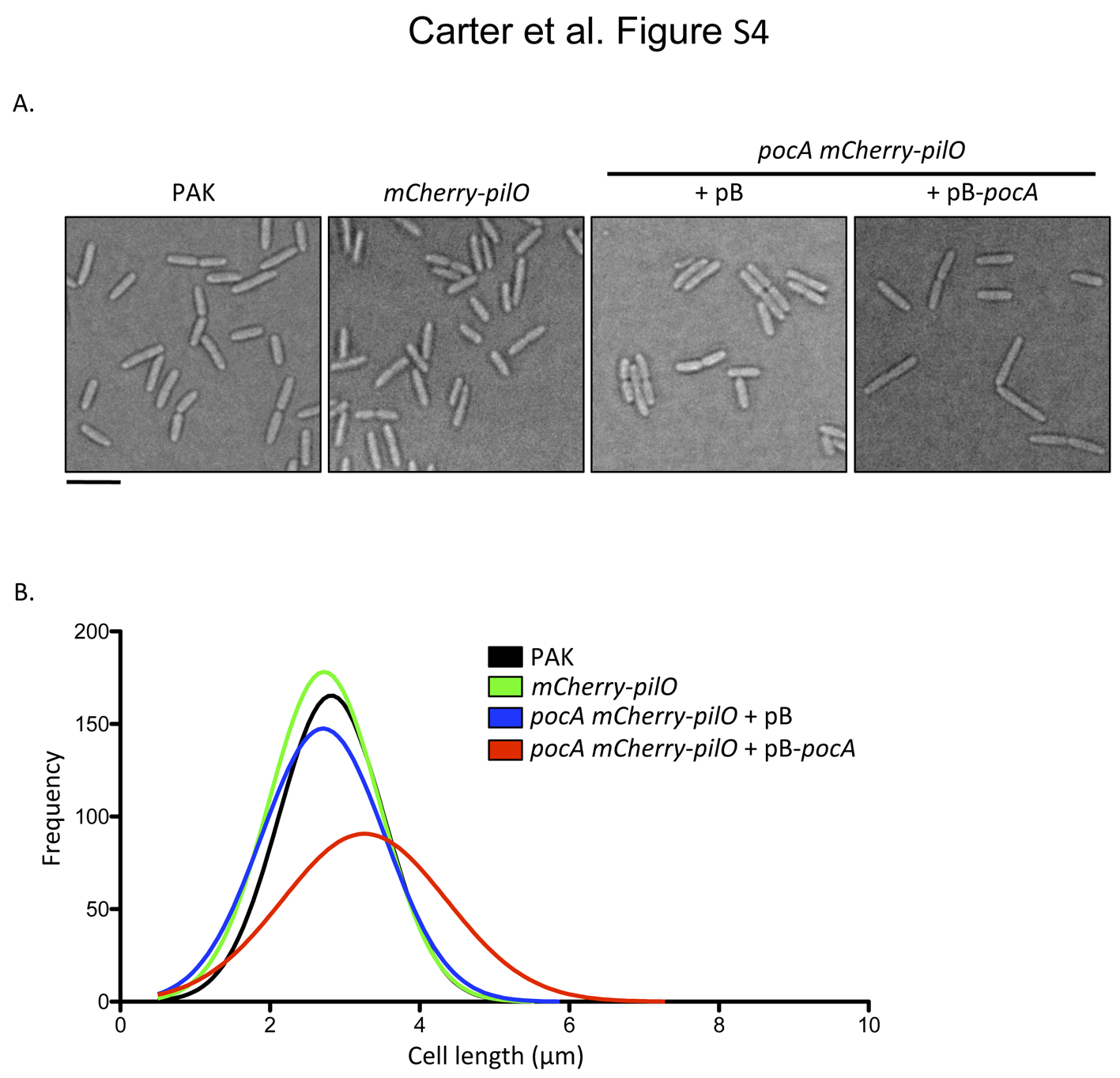

Supplement: FIG S4 [file mbo001173172sf4.tif]
